# Adaptogenic Activity of Lyophilized Hydroethanol Extract of *Pandanus odoratissimus* in Swiss Albino Mice

**DOI:** 10.1155/2014/429828

**Published:** 2014-10-28

**Authors:** Prafulla P. Adkar, Pranita P. Jadhav, Shirishkumar D. Ambavade, V. H. Bhaskar, Tushar Shelke

**Affiliations:** ^1^Post Graduates Department of Pharmacology, JSPM's Jayawantrao Sawant College of Pharmacy and Research, University of Pune, Pune, Maharashtra 411028, India; ^2^Vinayaka Missions University, Sankari Main Road, NH-47, Ariyanoor, Salem, Tamil Nadu 636308, India; ^3^Department of Pharmaceutical Medicinal Chemistry, Gahlot Institute of Pharmacy, Plot No. 59, Sector No. 14, Koparkhairane, Navi-Mumbai, Maharashtra 400709, India; ^4^JSPM's Charak College of Pharmacy and Research, University of Pune, Pune, Maharashtra 412207, India

## Abstract

*Background*. The leaves of *Pandanus odoratissimus* Linn have been widely used in Ayurveda to treat a variety of common and stress related disorders. In the present investigation, hydroethanol extract of leaves of *Pandanus odoratissimus* Linn (LEPO) were evaluated for antistress activity in normal and stress induced mice. Furthermore, the extract was studied for nootropic (adaptogenic) activity in mice and *in vitro* antioxidant potential to correlate with its adaptogenic and antistress activity. LEPO (100 and 200 mg/kg p.o) was evaluated against forced swimming endurance stress test, anoxia stress tolerance and immobilization stress and chronic cold resistant stress tests, and biomarkers (serum glucose, Corticosterone, WBC, RBC, and DLC count) to assess the antistress activity in mice. *Withania somnifera* (WS) (100 mg/kg p.o) was selected as reference standard. The parameters like anoxia stress tolerance time were recorded in anoxia stress and estimation of biochemical marker levels and determination of organs weight were carried out in immobilization stress models. *Results*. Concomitant treatment with LEPO 200 mg/kg significantly increased in anoxia stress tolerance time. Dose dependent significant reduction in serum glucose, corticosterone, and WBC, RBC, and DLC was observed in immobilisation stress model as compared to stressed group. LEOP 200 mg/kg and WS 100 mg/kg significantly reversed/inhibited the stress induced changes in these parameters. The results from the present study indicate that these values also express that dose dependent significant adaptogenic activity in stressed animals. *Conclusion*. The present study provides scientific support for the antistress (adaptogenic) and nootropic activities of lyophilized hydroethanol extract of *Pandanus odoratissimus* Linn and substantiate the traditional claims for the usage of Pandanus in stress induced disorders.

## 1. Introduction

According to the report of WHO, approximately 450 million people suffer from mental or behavioral disorders like stress. This amounts to 12.3% of the global burden of disease and is predicted to rise up to 15% by 2020. It is estimated that 75–90% of visits to primary care physicians are related to stress either acutely or because of chronic problems associated with stress. An October 2008 American Psychological Association (APA) press release on stress in America claimed that 8 of 10 Americans cite the economy as a significant source of stress, up from 66 percent six months earlier. In June 2008, more people were reporting symptoms associated with stress compared to the previous year, with nearly half polled indicating that stress had increased in the past years [1].

Stress is nonspecific response of the body known to alter the physiological homeostasis of the organism resulting in various neuronal, endocrine, and visceral dysfunctions. The ability to develop and maintain resistance against a variety of stressors encountered in human life is crucial for survival [2].

If stress increases the organism may become diseased. Stress is one of the basic factors which cause a number of diseases such as atherosclerosis, coronary heart disease, aging, and liver disease [1]. The desire to control the coping mechanisms has led to the origin of the science of adaptation [2].

Physiologists define stress as how the body reacts to a stressor, real or imagined, a stimulus that causes stress. Acute stressors affect an organism in the short term; chronic stressors over the longer term selye researched the effects of stress.

If exhaustion is extended, long term damage may result as the body and the immune system are exhausted and function is impaired resulting in decompensation. The result can manifest itself in obvious illnesses such as ulcers, depression, diabetes, and trouble with the digestive system or even cardiovascular problems, along with other mental illnesses [3].

Adaptogens are substances that help organisms to adapt to unfavorable stressful conditions, which could be physical, chemical, biological, or mental conditions. Some pioneer researchers in this field put forth specific criteria that need to be fulfilled to qualify as an adaptogens, which include ability to produce a nonspecific response (i.e., increases resistance against multiple physical, chemical, or biological stressors); it brings any dysfunctioning body system back into balance and must be innocuous, that is, must not influence normal body functions more than required. Adaptogens could be synthetic or natural substances. However, most researches on adaptogens have focused on natural substances (specifically plants), and the term “phytoadaptogens” is now commonly used for adaptogens of plant origin [2].

The Ayurvedic plant* Pandanus odoratissimus* Linn belonging to the family Pandanaceae Pandanus comprises 500–600 species and is distributed mainly in subtropical and tropical regions; out of these, 36 species have been recorded in India. It is widely distributed in India over coastal districts of Orissa (especially in Ganjam), Andhra Pradesh, Tamil Nadu, and to some extent in parts of Uttar Pradesh.

Preliminary phytochemical analysis positive tests for alkaloids, steroids, carbohydrates, phenolic compounds, glycosides, proteins and amino acids, and so forth [4].* Pandanus odoratissimus* contain the active chemical constituents which serve the medicinal properties: 2-phenyl ethyl alcohol, 2-phenyl ethyl methyl ether, terpinen-4-ol, 3-hydroxy-2-isopropenyl-dihydrobenzofuran-5-carboxylic acid methyl ester, 3-methyl-3-buten-1yl acetate, 3-Methyl-3-1 YL-Cinnamate, 3-methyl-3-buten-1yl-acetate-3-methyl-2-buten-1-yl cinnamate, 3,4-bis(4 hydroxy-3-methoxy benzyl) tetrahydrofuran, *α*-terpineol, *β*-carotene, *β*-sitosterol, benzyl-benzoate, pinoresinol, germacrene B, vitamin C, vinidine, tangerine, 5,8-hydroxy-7-methoxy-flavon. The leaves contain the pyridine alkaloids, pandamarilactone-1 (C_18_H_23_NO_4_), pandamarilactone-31 (C_19_H_25_NO_4_), and pandamarilactone-32 (C_18_H_21_NO_3_). The aroma compound 2-acetyl-1-pyrollidine hasbeen identified from the volatile oil of the leaf [5]. Lignans andbenzofurans have been isolated from roots of* Pandanus odoratissimus* [6, 7].

The evaluation of these plants and of their active natural principles is a logic way of searching for new drugs to treat this disease.* Pandanus odoratissimus* is said to be restorative, indolent, promoting a feeling of well-being, and acting as a counter to tropical climates. Ayurvedic science has found the medicinal action of essential oil yielded by the screw pine's highly scented flowers to be useful in headaches and earaches and as a liniment for rheumatic pains.

It may be chewed as a breath sweetener or used as a preservative in foods. It is also believed to have health-related properties, including antiviral, antiallergy, antiplatelet, anti-inflammatory, antioxidant, and anticancer action.* Pandanus odoratissimus *is said to be restorative, indolent, promoting a feeling of well-being, and acting as a counter to tropical climates [8].

The leaves of the plant have been mentioned valuable in leprosy, scabies, leucoderma, cephalalgia, coxalgia and otalgia, wounds, ulcers and colic, antioxidants [9]. The oil of the male flowers is considered stimulant and antispasmodic and is administered for headache and rheumatism [10].

## 2. Materials and Methods

### 2.1. Collection and Selection of Plant Material Preparation of Extract

**Figure 1:**
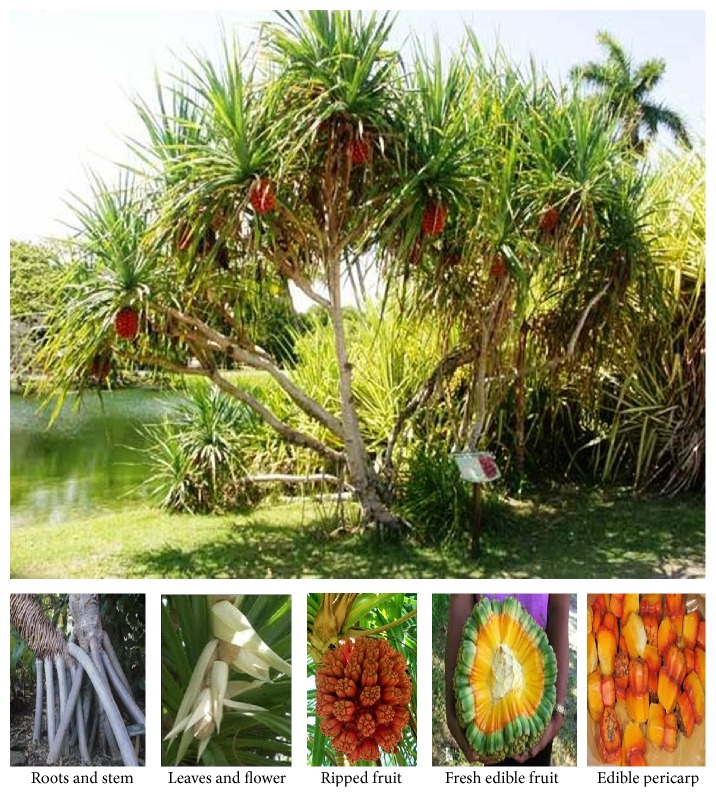
Plant monograph of* Pandanus odoratissimus *Linn (Kewda).

Fresh “Kewda” (*Pandanus odoratissimus* Linn) medicinal plants were collected from the surroundings of Lonavala Hill Station, Pune, Maharashtra (India), in April 2009 (Figure 1) and authenticated by the Botanical Survey of India, Pune, Maharashtra, India (voucher number MIM_PPA-18). Selected plant was powdered and material was separately weighed and used for extraction by the basis of polarity of solvent [6, 11–14]. The concurrent extraction (maceration) of powdered plant material (1 Kg) was soaked in 2.5 litres of (99.9% Ethanol + Distilled Water) (60 : 40) hydroethanol for 7 days at room temperature. After 7 days, the hydroethanol soluble materials of both plants were filtered off separately [10, 15].

### 2.2. Subfraction Lyophilisation of Hydroethanol Extract

They were filtered and kept at room temperature for cooling. Aqueous extract was freezed under −70°C for three days and then lyophilized. 1 g of lyophilized* Pandanus odoratissimus *Linn (LEPO) is equivalent to 2.01 g of dried* Pandanus odoratissimus* Linn hypocotyls. Lyophilized plant extract was diluted in distilled water to obtain a dose of 1 g/kg b.w. This dose has been proved to be optimal in a dose-dependent study [11, 12].

### 2.3. Selection of Experimental Animals

Albino mice (Swiss strain) of either sex weighing between 18 and 25 g were procured from central animal house serum Institute, Hadapsar, Pune, for experimental purpose. After procuring, the animals were acclimatized for seven days under standard husbandry condition with 12 : 12 hours light or dark cycle.

Healthy Swiss Albino mice the chosen experimental animals were maintained in our animal house (12 : 12 dark: light cycle), with adequate ventilation and hygienic conditions, maintained with normal pelleted diet (NUTRIVATE life science, Sinhagad Road, Pune, Baramati Agro Ltd. BVQI certified company) and water* ad libitum. *A group of animals were housed in polypropylene cage, paddy husk bed covered with stainless steel wire mesh with provision for water and feed. After obtaining prior permission from Institutional Animal Ethical Committee (IAEC) Protocol approval number JSCOPR/IAEC/02/2008-09, all animals' studies were performed in accordance with guidelines of “Committee for the Purpose of Control and Supervision of Experiments on Animals” (CPCSEA), Ministry of environment and Forest, Government of India.

### 2.4. Drugs and Chemicals Used

Drugs and chemicals used were as follows:

- marketed sample of* Withania somnifera*; Ashwagandha, 60 Caplets Himalaya Herbal Healthcare, Bangalore, India;
- marketed sample of* Pandanus odoratissimus*; Kewra Water (Kewda Water),* Top-Op*; Spice of India, India.

### 2.5. Acute Toxicity Study

Acute oral toxicity study for the proprietary formulation was carried out using OECD guideline-425 (modified, adopted March 23, 2006), the sequential test that uses a maximum of five animals. A test dose of 2000 or exceptionally 5000 mg/kg may be used in situation where experiment has information indicating that the test material is likely to be nontoxic.

The test procedure minimizes the number of animals required to estimate the oral acute toxicity of a chemical and in addition estimation of LD_50_, confidence intervals. The test also allows the observation of signs of toxicity and can also be used to identify chemicals that are likely to have low toxicity [11, 12].

As suggested, after acclimatization of animals for 4-5 days, study was carried out as follows. Healthy, young adult Albino Swiss mice (18–25 gm), nulliparous, and nonpregnant were used for this study. Food, but not water, was withheld for 3-4 hours and further 1-2 hours after administration of sample under study. One animal was received test drug (Plant extract) by oral route. Since this first test animal survived, four other animals were dosed (orally) at subsequent days, so that a total of five animals were tested.

Animals were observed individually at least every 5 minutes once during first 30 minutes after dosing, periodically at 2 hrs during the first 24 hours (with special attention during the first four hours), and daily thereafter, for a total of 14 days (Table 1).

## 3. Experimental Pharmacology

### 3.1. Scheduled Drug Treatments

Swiss albino mice (18–25 g) were for forced swim endurance stress test. The animals were divided into 5 groups consisting of 6 animals in each group. Group I was treated with saline water (1 mL). Group II was taken as a standard and was with treated with WS (100 mg/kg p.o). Group III was treated with marketed preparation of* Pandanus odoratissimus *Linn. (PO) (1 mL/kg) and Groups IV and V were taken as test groups and treated with (100 mg/kg and 200 mg/kg p.o) of the LEPO, respectively, for the all models,* namely*, forced swimming endurance stress test, anoxic stress tolerance test, and chronic cold resistant stress test.

### 3.2. Forced Swimming Endurance Stress Test

Swiss albino mice (18–25 g) were used for forced for forced swim endurance stress test. The animals were divided in to 5 groups and all were treated as mentioned above in scheduled drug treatments.

The drug treatment was given continuously for seven days (same dose daily). On the 8th day the mice were subjected to swimming stress by keeping them in polypropylene tank of dimension 37 × 37 × 30 cm filled with water to a height 25 cm. Mice were allowed to swim till complete exhaustion and the end point was taken when animal starting drowning. The mean swimming time for each group was calculated. The blood was collected through retroorbital puncture to estimate biochemical parameters like Serum glucose and Corticosterone and blood cells [1, 16].

### 3.3. Anoxic Stress Tolerance Test

Swiss albino mice (18–25 g) were used for forced for anoxic stress tolerancetest. The animals were divided into 5 groups and all were treated as mentioned above in scheduled drug treatments.

Conical flasks of 250 mL capacity were used for the study. These flasks were made airtight using rubber cork before beginning the experiment. Each animal was kept in the airtight vessel and time was noted using a stopwatch. The moment an animal showed first convulsion, it was removed immediately from the vessel and resuscitated if needed. The time duration from the entry of the animal in the hermetic (conical flask) vessel to the appearance of the first convulsion was taken as the time of “anoxic stress tolerance.” The mean time to convulsion was recorded and animal was removed at onset of convulsion. The blood was collected through retroorbital puncture to estimate biochemical parameters like serum glucose and Corticosterone and blood cells [1, 17].

### 3.4. Chronic Cold Resistant Stress Test

Swiss albino mice (18–25 g) were used for forced for chronic cold resistant stresstest. The animals were divided into 5 groups and all were treated as mentioned above in scheduled drug treatments.

The drug treatment was given continuously for seven days (same dose daily). In Each animals were subjected to cold stress by exposing them to 4 ± 10°C daily for 2 hours. The extracts were administered daily once to respective groups. This procedure was repeated for a period of 7 days. Blood was collected through orbital plexus under light ether anesthesia to estimate biochemical parameters like blood glucose, corticosteroid, and blood cell count (RBC and DLC). Animals were sacrificed by giving excess euthanasia [18, 19].

### 3.5. Statistical Analysis

All values were expressed as mean ± standard error of the mean. Result was analyzed statistically by using one-way ANOVA followed by Dunnett's multiple comparison test against the respective control. *P* ≤ 0.05 were considered to be significant.

## 4. Results

In swimming test model, seven day treatment increased swimming time to 125, 133, 145 by test drug and 416 by Withania sominifera as compare to control group 113 sec. Test Groups III, IV, and V swam for 125, 133, and 145 sec, respectively. When swimming time of Groups III, IV, and V was compared with Group I, it showed significant (*P* < 0.01) rise in the swimming time. 200 mg/kg LEPO treated animals showed reading very close to 100 mg/kg of WS treated animals. Consequently higher dose of LEPO is more potent with respect to lower dose of extract. Both the doses were able to increase the swimming endurance when compared with animals who received normal saline water (Table 2).

### 4.1. Biochemical Parameter

On 8th day level of glucose in serum was found to be in control group (I) 141.6 ± 1.6. In the standard group which is treated with WS the serum glucose level was found to be 98.3 ± 4.4 mg/dL, and the test control groups (III, IV and V) treated with PO, LEPO (100 mg/kg) and LEPO (200 mg/kg) serum glucose level were showed the 105 ± 2.8, 110 ± 1.0 and 131.6 ± 1.6 simultaneously. The level of glucose was increased during the time of stress but it came down due to pretreatment of LEPO.

The level of Corticosterone in serum (on 8th day) was recorded as 140 mg/dL for control group for control group-I, who received by saline water. and the group-II treated with WS (withania somnifera) was found to have the 107.3 ± 6.3 mg/dL level of Corticosterone in serum.

Groups III, IV, and V which were treated with marketed preparation of PO (*Kewda water*), LEPO 100, and 200 mg.kg, respectively, demonstrate the Corticosterone level in plasma 123.6 ± 1.6, 126.6 ± 3.3, 126.6 ± 1.6 mg/dL, respectively; marketed sample of PO (*Kewda water*) showed significant decrease in the level of Corticosterone (Table 4).

### 4.2. Hematological Parameters

On 8th day all animal groups hematological parameters were checked the group-I, served as normal control treated with saline water, group-II, served as slandered control treated with WS, in the RBC count parameter were found as 9.3 and 7.53 million/cumm simultaneously. The RBC count of Groups III, IV, and V was found to be 8.53, 7.56, and 7.93 million/cumm with respect to Group I in which it reduced due to pretreatment of extracts. The neutrophils count of animals of Groups I, II, III, and IV was as found to be 13.3, 10.3, 11.29, 12.3, and 12.3%, respectively. The neutrophils count of Groups II, III, and IV reduced due to pretreatment of extracts with respect to Group I. The lymphocytes count of animals of Groups I, II, III, IV, and V was found as 59.66, 55.33, 56.98, 59.6, and 58.33%, respectively. The lymphocyte count of Groups III and IV reduced due to pretreatment of extracts with respect to Group I. The eosinophil count of animals of Group I, II, III, IV, and V was found as 3.3, 1.4, 2.1, 2.6, and 1.5, respectively. The eosinophil count of groups III and IV reduced due to pretreatment of extracts with respect to Group I. The monocyte count of animals of Groups I, II, III, and IV was found as 3, 1, 1.6, 1.3, and 1.3%, respectively. The monocyte count of Groups IV and V reduced due to pretreatment of extracts with respect to Group I. Consequently forced swimming endurance test significantly altered the hematological parameters, that is, decreased RBC and DLC counts. Pretreatment with WS and LEPO significantly (*P* < 0.01) inhibited the stress induced changes in these parameters. On the other hand these values also express that higher dose of LEPO was very effective, but both the doses of test drug had shown significant adaptogenic activity (Table 3).

In anorexia stress tolerance test Group I (control) treated with saline water (1 mL) was kept in hermetic vessel to for 18.33 min. Group II taken as a standard treated with Withania somnifera survive in hermetic vessel for 69 min; Groups III, IV, and V animals survive in hermetic vessel 41, 33, and 28 min, respectively. The anoxia tolerance test was determined by taking the appearance of convulsion as end point. The LEPO at two doses (100 and 200 mg/kg) and PO (*Kewada water*) at dose (1 mL) showed significant (*P* < 0.01) increasing tolerance stress time in 8th day as compared with the control (Table 5).

### 4.3. Biochemical Parameters for Anorexia Stress Tolerance Test

On 8th day level of glucose in serum control group (I) was found to be 120.33; in the standard group which is treated with WS the serum glucose level it was found to be 81.6 ± 1.6 mg/dL. In the test drug groups (III, IV, and V), 95 ± 2.8, 111.6 ± 1.6, and 101.33 ± 0.6, the level of glucose was increased during the time of stress but it came down due to pretreatment of LEPO. The level of Corticosterone in serum (on 8th day) was recorded as 141 ± 1.6 mg/dL for control group who served by Saline water (1 mL). Group-II, treated with WS, and corticosterone level were found as the 85 ± 2.8 mg/dL level of Corticosterone in serum. Groups III, IV, and V which were treated with marketed preparation of PO (*Kewda water*) and LEPO 100 and 200 mg/kg, respectively, demonstrate the Corticosterone level in plasma 103.3 ± 1.6, 118.3 ± 4.4, and 110 ± 5.7 mg/dL, respectively. 200 mg/kg of LEPO and marketed sample of* Pandanus odoratissimus *Linn (PO;* Kewda water*) showed significant decrease in the level of Corticosterone (Table 7).

### 4.4. Hematological Parameters for Anorexia Stress Tolerance Test

On 8th day RBC count of animals of Group I treated as control, Group II treated as a standard, Group III for marketed preparation PO (*Kewda water*), and Groups IV and V treated with LEPO was found as 10.3, 7.4, 7.5, 7.6, and 7.6 million/cumm, respectively. The RBC count of Groups III, IV, and V with respect to Group I reduced due to pretreatment of extracts. The neutrophils count of animals of Groups I, II, III, and IV was found as 16, 10.3, 10.6, 11.05, and 11.3%, respectively. The neutrophils count of Groups III and V reduced due to pretreatment of marketed sample and extracts with respect to Group I. The lymphocytes count of animals of Groups I, II, III, and IV was found as 66, 50.6, 53.6, 58, and 54.6%, respectively. The lymphocyte count of Groups III, IV, and V reduced due to pretreatment of marketed sample and extracts with respect to Group I. The eosinophil count of animals of Groups I, II, III, IV, and V was found as 3, 0.6, 1, 1.3, and 1.3, respectively. The eosinophil count of Groups II, III, and IV reduced due to pretreatment of PO (*Kewda water*) and extracts with respect to Group I. The monocyte count of animals of Groups I, II, III, and IV was found as 3.3, 1.3, 1.3, 1.6, and 1.6%, respectively. The monocyte count of Groups II, III, and IV reduced due to pretreatment of extracts with respect to Group I. Consequently anoxia stress tolerance test significantly altered the hematological parameters, that is, increased RBC and DLC counts. Pretreatment with marketed sample and extracts significantly (*P* < 0.01) inhibited the stress induced changes in these parameters. On the other hand, these values also express that higher dose of LEPO was very effective, but both the doses of test drug had shown significant adaptogenic activity (Table 6).

### 4.5. Biochemical Parameters for Chronic Cold Resistance Test

On 8th day level of glucose in serum control group (I) was found to be 116 ± 1.6. In the standard group which is treated with WS the serum glucose level was found to be 70 ± 3.1 mg/dL. In the test drug Groups III, IV, and V, 101.6 ± 1.6, 112.3 ± 1.4, 116.6 ± 3.3, the level of glucose was increased during the time of stress but it came down due to pretreatment of PO (*Kewda water*) and LEPO.

The level of Corticosterone in serum (on 8th day) was recorded as 143.3 ± 1.6 mg/dL for control Group I Saline water (1 mL). Group II treated with WS was found to have the 104.6 ± 2.4 mg/dL level of Corticosterone in serum.

Groups III, IV, and V which were treated with marketed preparation PO (*Kewda water*) and LEPO 100 and 200 mg·kg, respectively, demonstrate the Corticosterone level in plasma 110 ± 5.7, 118 ± 1.6, and 113.3 ± 6.6 ng/dL, respectively. Marketed sample and LEPO showed significant decrease in the level of Corticosterone (Table 9).

### 4.6. Hematological Parameter for Chronic Cold Resistance Test

On 8th day in Group I treated as control and Group II treated as a standard with WS, the RBC count for animals was found as 9.6 and 7.4 million/cumm. The RBC count of Groups III, IV, and IV was found to be 7.5, 7.9, and 7.7 million/cumm with respect to Group I in which it reduced due to pretreatment of marketed sample and extracts. The neutrophils count of animals of Groups I, II, III, and IV was found as 13.3, 10.3, 10.6, 11.3, and 11.6%, respectively. The neutrophils count of Groups II, III, and IV reduced due to pretreatment of marketed sample and extracts with respect to Group I. The lymphocytes count of animals of Groups I, II, III, IV, and V was found as 61.6, 52, 52.3, 56, and 55%, respectively. The lymphocyte count of Groups III and IV reduced due to pretreatment of extracts with respect to Group I. The eosinophil count of animals of Groups I, II, III, IV, and V was found as 3.3, 1.3, 1.6, 1.6, and 1.3, respectively. The eosinophil count of Groups III and IV reduced due to pretreatment of extracts with respect to Group I. The monocyte count of animals of Groups I, II, III, and IV was found as 2.3, 1, 1.3, 1.3, and 1%, respectively. The monocyte count of Groups IV and V reduced due to pr-treatment of PO (*Kewda water*) and LEPO with respect to Group I. Consequently cold stress test significantly altered the hematological parameters, that is, decreased RBC and DLC counts. Pretreatment with WS and LEPO significantly (*P* < 0.01) inhibited the stress induced changes in these parameters. On the other hand these values also express that higher dose of LEPO was very effective, but both the doses of test drug had shown significant adaptogenic activity (Table 8).

## 5. Discussion

Stre**ss** alters the equilibrium of various hormones which have a significant impact on the immune response in general. The status of immune system immunosuppression versus immunopotentiation will depend upon the net effect of these changes. Stress has been shown to affect immune system functioning with both immunosuppression and immunopotentiation.

Plant adaptogens are smooth prostressor which reduces the reactivity of host defense system and decreases the damaging effect of various stressors due to increase of basal level mediators involved in the stress response [20].

The forced swimming is the most widely used method for assessing the antistress property of a compound. This paradigm is based on the observation that animal when forced to swim in water eventually assumed characteristics immobile posture, devoid of any activity. The appearance of immobility, therefore, reflects a state of tiredness, fatigue, and reduced stamina with the end point being the moment when the mice could not swim further and started drowning [3, 18].

The increased swimming time has been observed in mice pretreated with* Pandanus odoratissimus *with enhanced physical performances significantly longer than untreated (control) group and thus confirmed selected Indian medicinal plants having the adaptogenic nature.

During stress, blood glucose level increases which are found to be significantly reduced in* Pandanus odoratissimus* treated mice. Lowering of stress induced hyperglycemia is an indication of Antistress, adaptogenic activity of plant.

In response to stress, ACTH is released, which acts on the adrenal cortex to stimulate the synthesis and release of cortisol [21]. Increased plasma cortisol influences the mobilization of stored fat and carbohydrate reserves which in turn increased blood glucose level. The increased cortisol levels are reversed by antistress agents [21]. LEPOsignificantly decreases the stress induced elevated levels of cortisol and blood glucose level. The reference drug in this study, WS, also produced similar result. Stressed animals spleen contracts and releases the more amounts of blood cells (RBC's and WBC's) into circulations. (this stage in acute stress phenomenon) [1, 17]. The stress induced RBC and WBC count is decreased by the LEPO. Increased swim duration and decreased (this stage in chronic stress phenomenon) in RBC and WBC count in mice pretreated with* Pandanus odoratissimus* are similar to the changes produced by reference drug* Withania somnifera*.

Anorexia is a very sever stressor. All the body functions including cellular respiration depend on oxygen supply to them. Any lack of this vital element plays havoc on all body mechanisms. Increase in adaptation during this stress by any drug could be considered as its major antistress effect [3]. The result of the study demonstrates that the Pandanus odoratissimus extract significantly prolonged the meantime to convulsion, which therefore confirms its antistress property. Prolongation of meantime to convulsion could be a result of its powerful antioxidant and free radical scavenging activities [3, 18], the mechanism by which stress rises which enhances the activity of hypothalamo-hypophyseal axis (HPA) resulting in liberation of catecholamine and corticosteroids. The increase release of catecholamine leads to elevated levels of glucose. Stress induces adrenomedullary response in man. Adrenaline in turn stimulates *β*_2_ receptor on the pituitary gland causing greater release of ACTH, which can stimulate the adrenal medulla as well as cortex. Cortisol increases mRNA level in liver cells. Spleen constricts to release more blood cells (RBC and WBC) during stress, so their weights decrease in stress [22]. This stress induced changes were significantly reversed by the test extract at lower and higher doses. Experimental studies have confirmed the adaptogenic activity of* Pandanus odoratissimus*.

Cold stress is a psychogenic type [23]. Response to stress is highly contradictory with regard to blood sugar level. A significant hyperglycemia in cold restraint stress model was observed. Under stressful condition Corticosterone in mice will be secreted by adrenal cortex. Hyper secretion of Corticosterone helps the maintenance of internal homeostasis through the process of gluconeogenesis [16]. The treatment with the lyophilized hydroethanol extract of leaves of* Pandanus odoratissimus* significantly reduced the cold stress related hyperglycemias. The re-ducting the hyperactivity of hormonal secretions of adrenal cortex and it's beneficial to maintain homeostatic mechanism [1].

Stress also causes the alteration in hematological parameter like increase in RBC and WBC [1]. Pretreatment with lyophilized hydroethanol extract of leaves of* Pandanus odoratissimus* (LEPO) and reference drug* Withania somnifera* (WS) reduced the hematological parameters significantly [19].

## 6. Conclusion

LEPO at dose of 100 and 200 mg/kg and the marketed sample have shown significantly that they possess the ability to prevent alteration due to stress. The above results are promising and further dose variant studies would be helpful in substantiating the claim of adaptogenic action of this drug.

Thus with all above results it can be claimed and concluded that the marketed and the lyophilized hydroethanol extract of leaves of* Pandanus odoratissimus *Linn (LEPO) have adaptogenic property.

## Figures and Tables

**Table 1:** Observation (AOT) report on acute oral toxicity test.

Sample: hydroethanol extract of *Pandanus odoratissimus *				
Test guideline OECD 425 (modified October 2006) limit test				

Animal used: Albino Swiss mice				
Sex: female albino Swiss mice				
Weight range: 18–25 gm				
Route of drug administration: per oral				
Dose: 2000 mg/kg				
Fasted for 3-4 hrs (before experimentation) and 1-2 hrs (after experimentation)				

No. of animal used	Date of testing			

3				
3				

Observations	1/2 hr	1 hr	2 hr	4 hr

Awareness	4	4	4	4
Mood	0	0	0	0
Motor activity	6	6	6	6
Neurological profile				
(a) Central excitation	0	0	0	0
(b) Muscle tone	4	4	4	4
(c) Motor incoordination	0	0	0	0
(d) Reflexes	4	4	4	4
(f) Autonomic profile	4	4	4	4
Screening sign				
(a) Urination	0	0	0	0
(b) Salivation	0	0	0	0
General sign				
Skin color	4	4	4	4
Heart rate	4	4	4	4
Respiratory rate	4	4	4	4
Death	—	—	—	—

Inference: at 2000 mg/kg sample did not produce any observable toxic effects during entire duration of study and all animals survived 14 days of observation.				

Conclusion: LD_50_ of test is 2000 mg/kg.				

**Table 2:** Effect of LEPO on swimming survival time (sec) for forced swimming endurance test in Swiss Albino mice for 7-day study.

Groups	Treatment, dose (mg/kg body wt.) p.o	Swimming survival time (sec)
Group I; negative control	Saline water (1 mL)	113 ± 2.9
Group II; positive control	WS; (100)	416.6 ± 3.8^**^
Group III; test control	PO; (*Kewda water*) (0.5)	125.6 ± 3.2^*^
Group IV; test control	LEPO (100)	133.3 ± 2.4^**^
Group V; test control	LEPO (200)	145 ± 3.8^**^

*n* = 6 animals in each group.

WS: *Withania somnifera*; LEPO: lyophilized hydroethanol extract of leaves of *Pandanus odoratissimus* Linn; PO; (*Kewda water*): marketed preparation of *Pandanus odoratissimus*.

Values are mean ± S.E.M. expressed of each group. Data analysis was performed using one-way ANOVA followed by Dunnett's multiple comparison test against the respective control. ^*^ *P* < 0.5, ^**^ *P* < 0.01.

**Table 3:** Effect of LEPO on hematological parameters for forced swimming endurance test in Swiss Albino mice for 7-day study.

Groups	Treatment, dose mg/kg body wt. p.o	Hematological parameters in values are mean ± S.E.M.				
		RBCS	Neutrophils	Lymphocytes	Monocytes	Eosinophils
Group I; negative control	Saline water (1 mL)	9.3 ± 0.3	13.3 ± 0.6	59.66 ± 0.3	3.00 ± 0.5	3.3 ± 0.3
Group II; positive control	WS; (100)	7.53 ± 0.3^**^	10.33 ± 0.3^**^	55.33 ± 0.3^**^	1.00 ± 0.0^*^	1.3 ± 0.3^**^
Group III; test control	PO; (*Kewda water*) (0.5)	8.53 ± 0.1	11.29 ± 0.3^*^	56.98 ± 0.3^**^	1.40 ± 0.3^*^	1.5 ± 0.3^**^
Group IV; test control	LEPO; (100)	7.56 ± 0.03^**^	12.33 ± 0.3	59.66 ± 0.6	1.60 ± 0.3	2.0 ± 01^*^
Group V; test control	LEPO; (200)	7.93 ± 0.1^**^	13.01 ± 0.3	58.00 ± 0.5^*^	1.30 ± 0.3^*^	1.6 ± 0.3^**^

*n* = 6 animals in each groups.

WS: *Withania somnifera*; LEPO: lyophilized hydroethanol extract of leaves of *Pandanus odoratissimus* Linn; PO; (*Kewda water*): marketed preparation of *Pandanus odoratissimus*.

Values are mean ± S.E.M. expressed of each group. Data analysis was performed using one-way ANOVA followed by Dunnett's multiple comparison test against the respective control. ^*^ *P* < 0.5, ^**^ *P* < 0.01.

**Table 4:** Effect of LEPO on biochemical parameters in forced swimming endurance test in Swiss Albino mice for 7-day study.

Groups	Treatment, dose mg/kg body wt. p.o	Reducing sugar	Corticosterone
Group I; negative control	Saline water (1 mL)	141.6 ± 1.6	140.0 ± 1.6
Group II; positive control	WS; (100)	98.3 ± 4.4^**^	107.3 ± 6.3^**^
Group III; test control	PO; (*Kewda water*) (0.5)	105 ± 2.8^**^	123.6 ± 1.6^*^
Group IV; test control	LEPO; (100)	131.6 ± 1.6	126.6 ± 3.3^*^
Group V; test control	LEPO; (200)	110 .0 ± 1.0^**^	126.6 ± 1.6^*^

*n* = 6 animals in each group.

WS: *Withania somnifera*; LEPO: lyophilized hydroethanol extract of leaves of *Pandanus odoratissimus* Linn; PO; (*Kewda water*): marketed preparation of *Pandanus odoratissimus*.

Values are mean ± S.E.M. expressed of each group. Data analysis was performed using one-way ANOVA followed by Dunnett's multiple comparison test against the respective control. ^*^ *P* < 0.5, ^**^ *P* < 0.01.

**Table 5:** Effect of LEPO on duration of anorexic stress tolerance for anorexic stress tolerance test in Swiss Albino mice for 7-day study.

Groups	Treatment, dose mg/kg body wt. p.o	Duration of anoxic stress tolerance (min) mean ± SE
Group I; negative control	Saline water (1 mL)	18.33 ± 0.6
Group II; positive control	WS; (100)	69.05 ± 1.2^**^
Group III; test control	PO; (*Kewda water*) (0.5)	41.83 ± 2.0^**^
Group IV; test control	LEPO; (100)	28.67 ± 0.8^**^
Group V; test control	LEPO; (200)	33.83 ± 0.9^**^

*n* = 6 animals in each group.

WS: *Withania somnifera*; LEPO: lyophilized hydroethanol extract of leaves of *Pandanus odoratissimus* Linn; PO; (*Kewda water*): marketed preparation of *Pandanus odoratissimus*.

Values are mean ± S.E.M. expressed of each group. Data analysis was performed using one-way ANOVA followed by Dunnett's multiple comparison test against the respective control. ^*^ *P* < 0.5, ^**^ *P* < 0.01.

**Table 6:** Effect of LEPO on hematological parameters for anorexia stress tolerance test in Swiss Albino mice for 7-day study.

Groups	Treatment, dose mg/kg body wt. p.o	Hematological parameters in values are mean ± S.E.M.				
		RBCS	Neutrophil	Lymphocyte	Monocyte	Eosinophils
Group I; negative control	Saline water (1 mL)	10.3 ± 0.3	16 ± 1	66	3.3 ± 0.3	3 ± 0.5
Group II; positive control	WS; (100)	7.4 ± 0.03^**^	10.3 ± 0.3^**^	50.6 ± 0.6^**^	1.3 ± 0.3^**^	0.6 ± 0.3^**^
Group III; test control	PO; (*Kewda water*) (0.5)	7.5 ± 0.1^**^	10.6 ± 0.3^**^	53 ± 1.5^**^	1.3 ± 0.3^**^	1 ± 0.0^*^
Group IV; test control	LEPO; (100)	7.6 ± 0.1^**^	11 ± 0.5^**^	58.3 ± 1.2^*^	1.6 ± 0.3^*^	1.3 ± 0.3^*^
Group V; test control	LEPO; (200)	7.6 ± 0.1^**^	11.3 ± 0.3^**^	54.6 ± 1.6^**^	1.6 ± 0.3^*^	1.3 ± 0.3^*^

*n* = 6 animals in each group.

WS: *Withania somnifera*; LEPO: lyophilized hydroethanol extract of leaves of *Pandanus odoratissimus* Linn; PO; (*Kewda water*): marketed preparation of *Pandanus odoratissimus*.

Values are mean ± S.E.M. expressed of each group. Data analysis was performed using one-way ANOVA followed by Dunnett's multiple comparison test against the respective control. ^*^P < 0.5, ^**^ *P* < 0.01.

**Table 7:** Effect of LEPO on biochemical parameters in anorexia stress tolerance test in Serum in Swiss Albino mice for 7-day study.

Groups	Treatment, dose mg/kg body wt. p.o	Reducing sugar	Corticosterone
Group I; negative control	Saline water (1 mL)	120.33 ± 0.3	141 ± 1.6
Group II; positive control	WS; (100)	81.6 ± 1.6^**^	85 ± 2.8^**^
Group III; test control	PO; (*Kewda water*) (0.5)	95 ± 2.8^**^	103.3 ± 1.6^**^
Group IV; test control	LEPO; (100)	111.6 ± 1.6^*^	118.3 ± 4.4^**^
Group V; test control	LEPO; (200)	101.33 ± 0.6^**^	110 ± 5.7^**^

*n* = 6 animals in each group.

WS: *Withania somnifera*; LEPO: lyophilized hydroethanol extract of leaves of *Pandanus odoratissimus* Linn; PO; (*Kewda water*): marketed preparation of *Pandanus odoratissimus*.

Values are mean ± S.E.M. expressed of each group. Data analysis was performed using one-way ANOVA followed by Dunnett's multiple comparison test against the respective control. ^*^ *P* < 0.5, ^**^ *P* < 0.01.

**Table 8:** Effect of LEPO on hematological parameters cold endurance test in Swiss Albino mice for 7-day study.

Groups	Treatment, dose mg/kg body wt. p.o	Hematological parameters in values are mean ± S.E.M.				
		RBCS	Neutrophil	Lymphocyte	Monocyte	Eosinophils
Group I; negative control	Saline water (1 mL)	9.6 ± 0.8	13.6 ± 0.3	61.6 ± 1.6	3.3 ± 0.3	2.3 ± 0.3
Group II; positive control	WS; (100)	7.4 ± 0.03	10.3 ± 0.3^**^	52.3 ± 1.1^**^	1.3 ± 0.3^**^	1^**^
Group III; test control	PO; (*Kewda water*) (0.5)	7.5 ± 0.17	10.6 ± 0.6^**^	52 ± 1.4^*^	1.3 ± 0.3^**^	1 ± 0.6^*^
Group IV; test control	LEPO; (100)	7.9 ± 0.05	11.6 ± 0.3^*^	56 ± 1.1^*^	1.6 ± 0.3^*^	1.3 ± 0.3
Group V; test control	LEPO; (200)	7.7 ± 0.1	11.3 ± 0.3^**^	55 ± 0.5^*^	1.6 ± 0.3^*^	1.3 ± 0.3

*n* = 6 animals in each group.

WS: *Withania somnifera*; LEPO: lyophilized hydroethanol extract of leaves of *Pandanus odoratissimus* Linn; PO; (*Kewda water*): marketed preparation of *Pandanus odoratissimus*.

Values are mean ± S.E.M. expressed of each group. Data analysis was performed using one-way ANOVA followed by Dunnett's multiple comparison test against the respective control. ^*^ *P* < 0.5, ^**^ *P* < 0.01.

**Table 9:** Effect of LEPO on biochemical parameters in cold endurance test in Swiss Albino mice for 7-day study.

Groups	Treatment, dose mg/kg body wt. p.o	Reducing sugar	Corticosterone
Group I; negative control	Saline water (1 mL)	116 ± 1.6	143.3 ± 1.6
Group II; positive control	WS; (100)	70 ± 3.1^**^	104.6 ± 2.4^**^
Group III; test control	PO; *(Kewda water) *(0.5)	101.6 ± 1.6^**^	110 ± 5.7^**^
Group IV; test control	LEPO; (100)	116.6 ± 3.3^**^	118 ± 1.6^**^
Group V; test control	LEPO; (200)	112.3 ± 1.4^**^	113.3 ± 6.6^**^

*n* = 6 animals in each group.

WS: *Withania somnifera*; LEPO: lyophilized hydroethanol extract of leaves of *Pandanus odoratissimus* Linn; PO; (*Kewda water*): marketed preparation of *Pandanus odoratissimus*.

Values are mean ± S.E.M. expressed of each group. Data analysis was performed using one-way ANOVA followed by Dunnett's multiple comparison test against the respective control. ^*^ *P* < 0.5, ^**^ *P* < 0.01.
